# The Punishment of Abortion-Mongers

**Published:** 1887-06

**Authors:** 


### THE PUNISHMENT OF ABORTION-MONGERS.

Criminal abortion is so common in Atlanta, and so little effort is made by the authorities to suppress it, that we publish the following from the *Medical Press*, of London, with the hope that it will serve a good purpose: "It is comforting to find that the individuals who derive a precarious income by procuring abortion are occasionally brought within the reach of the law and receive condign punishment. In London two persons have been sentenced each to fifteen years' penal servitude last week for having performed an illegal operation on a young woman which resulted in her death, and almost at the same time three other persons received five years each for a similar operation at Leeds with the same untoward result. One's satisfaction, however, is lessened by the fact that the punishment only appears to attend such operations when they prove fatal to their victim. It is exceedingly rare that any cases come before the courts when the *attempt*, successful or otherwise, is made the subject of the charge. Although the nature of the offense binds all the parties concerned to secrecy, a little careful observation on the part of the police would often suffice to bring home the offense to people who are too skillful or too cunning to allow themselves to be committed on a charge of murder. If the police only displayed the same energy and detective ability in the search after this disreputable class of persons that they do, spasmodically, in the hunt after the comparative harmless fortune-tellers, it cannot be doubted that, even if not prevented, the calling would be rendered infinitely more hazardous than it appears to be at present. In an infinitesimal proportion of the cases only do the police get wind of the matter, and then, for the most part, by the intermediary of the coroner's officer. The indifference of the police in this respect is not creditable to the force, and calls for more stringent measures. For one *maladroit* who is caught in the meshes of the criminal law, hundreds, if not thousands, escape. Pills are openly advertised which claim to be possessed of the power of remedying the effect of sexual indiscretion, and no attempt is made to restrain their sale."

### ITEMS.

DR. T. S. HOPKINS, of Thomasville, was in Atlanta a few days ago.

THE ATLANTA UNIVERSITY for colored people has established a training school for nurses, which, we learn, is doing a good work.

AT the recent meeting of the Texas State Medical Association Dr. Samuel R. Burroughs was elected President and Dr. F. E. Daniel, of Austin, Secretary.

WE are in receipt of the May number of the *New Princeton Review*, published by A. C. Armstrong & Son, New York. It is a valuable publication, and must be read to be appreciated.

DR. A. G. WHITEHEAD, of Waynesboro, President of the Medical Association of Georgia, was in Atlanta a few days ago, and gave THE JOURNAL a pleasant call.

THE *Indiana Medical Journal* says that it "will risk its reputation on the assertion that the physician who charges only one dollar a visit usually gets all his services are worth, while he who does private practice on yearly contracts is a sodden ass whom 'twere base flattery to call a fool."

DR. D. G. BRINTON has retired from the *Medical and Surgical Reporter*, leaving it under the editorial management of Drs. N. A. Randolph and C. W. Dulles. This journal has been brought up to such a high standard by Dr. Brinton, and he is so well and favorably known by all professional readers in this country, that his name will prove a talisman for any other publication with which he may be associated in future.

A NUMBER of delegates to the American Medical Association have arranged to leave Atlanta at 1 o'clock p. m., June 5th, by the East Tennessee, Virginia and Georgia Railroad, arriving in Chicago via Cincinnati at 6:50 o'clock next evening. It is hoped that the delegation will be sufficiently large to justify running a sleeper through to Chicago. Those desiring sleeping-car berths and further information will address L. J. Ellis, Assistant General Passenger Agent, Atlanta, Georgia.

INTERNATIONAL CONGRESS ON INEBRIETY.—The Council of the English Society for the Study and Cure of Inebriety have completed arrangements for an International Medical Congress, to be held at Westminster Hall, London, July 5th and 6th, 1887. The object of this Congress is to present and discuss the problems of inebriety medically, and from a purely scientific standpoint, by the best authorities, thus laying the foundation for a broader and more exact study of this subject.

THE ECLECTIC MAGAZINE for June is an unusually fine number. Any one desiring to read the foreign literature of the day should subscribe for it. It is published by E. R. Pelton, 25 Bond street, New York.

THE study of "Some Illustrations of Napoleon and his Times," by John C. Ropes, which will appear in *Scribner's* for June, will contain a great many fine portraits and several caricatures. Another article, soon to be published in the same magazine, will complete the study.

IN the report of the proceedings of the Medical Association of Georgia, in the May number of THE JOURNAL, we failed to mention the able report on surgery from Dr. DeSaussure Ford, chairman of the section on surgery for the tenth district. The report was read by Dr. James M. Hull, of Augusta, and was referred to the Committee on Publication.

VAGINAL PRESSURE IN THE TREATMENT OF CHRONIC PELVIC DISEASE.—Dr. A. Reeves Jackson recently read a paper on this subject before the Chicago Gynæcological Society (*Journal American Medical Association*), in which he gives Dr. V. H. Taliaferro, of this city, credit for first bringing the subject to the attention of the profession, in a paper published in 1878, on "The Application of Pressure in Diseases of the Uterus." Dr. Jackson says: "I was much impressed by the stated results of the treatment, and determined to give it a trial. It seemed to promise a valuable substitute in some of the objectionable and uncertain methods of local treatment then and now in vogue, such as cauterization, local blood-letting, tents, intra-uterine medication, iodine painting, hot douches, etc. Since then I have used it in many cases of chronic pelvic disease, and am able to corroborate the favorable statements that have been made concerning its efficacy."

PEPTOGENIC MILK POWDER AS A MEANS OF PREDIGESTING COW'S MILK FOR FEEDING INFANTS.—At a recent meeting of the Atlanta Society of Medicine, Mr. James Carr, representing Fairchild Brothers & Foster, of New York, was invited to demonstrate the digestive powers and therapeutic adaptability of this preparation. The cow's milk, after being treated with the peptogenic powder, showed a close resemblance in appearance and taste to mother's milk. The addition of a few drops of hydrochloric acid showed by the character of the curd precipitated that the casein had partially been digested, and in its fine flocculent character resembled the curd formed in mother's milk under the same conditions.

THE BERGEON TREATMENT OF CONSUMPTION.—When we reflect that consumption annually numbers more victims than all the zymotic affections combined, and that more than one-twelfth of the deaths which occur every year are due to that dread disease, we do not wonder at the credulous enthusiasm with which the hasty reports of the value of the Bergeon treatment were received by countless unhappy patients and their sympathizing friends. We are amazed, however, that thinking physicians should allow themselves to be deceived into thinking that a degenerative process, seated deeply in the pulmonary tissues, could be arrested by pumping a few volumes of carbonic acid gas and sulphuretted hydrogen into the rectum. Of course, in cases complicated by intestinal ulceration, the anæsthetic effect of the former gas will lessen the pain, and consequently inspire the sufferer with new hope for the time being. In all other cases the Bergeon treatment is not only valueless, but positively injurious, as it arouses a hope which can never be realized, and which in a few weeks is rudely dispelled, leaving the patient dispirited and less able to withstand the ravages of the disease than at first.—*Medical Bulletin*.

FALSE HONORS—"TAIT'S OPERATION" NO MORE, BUT "BATTEY'S OPERATION."—Like the operation of ovariotomy, America is slowly but surely receiving the acknowledgment due her surgeons for the early pioneer work in abdominal surgery in the diseases of women. The reaction has set in, and Dr. Robert Battey, of Rome, Ga., is now accorded the honor of introducing to the profession the operation that has in it certain principles, which, if not abused in our enthusiasm, will mark one of the greatest epochs in the special field of surgery known as gynæcology. While the profession have much to admire in Mr. Lawson Tate, we must not forget to "give honor to whom honor is due."—*Homœopathic Journal of Obstet.*

NOTICE TO DELEGATES TO THE AMERICAN MEDICAL ASSOCIATION.—Mr. Fred. D. Bush, District Passenger Agent of the Louisville and Nashville Railroad, furnishes us the following: "Reduced rates to the Association will be made on the certificate plan; that is, that you must purchase your ticket to Chicago, paying therefor the regular one-way rate, and get a certificate from the agent selling you the same, stating the point from which sold, the route of the ticket, and the amount paid for it. When you arrive in Chicago, the Secretary of the Association will endorse on your certificate that you have been in attendance, and that will be authority for this company to sell you a return ticket at *one-third* the regular rate. The Louisville and Nashville Railroad offers delegates choice of two routes, via Nashville and Evansville, the Chicago Short-Line or via Nashville and Louisville with privilege of stopping over and visiting Mammoth Cave (America's greatest wonder) either going or returning. Through trains leave Atlanta daily at 7:50a. m., 5:50 p. m. and 11 p. m. The L. & N. is the short line to Chicago and makes the lowest rates. For maps, rates and other information write to Fred. D. Bush, D. P. A., Atlanta, Ga."

DOES IT FAVOR SAM?—The following is vouched for by a well-known South Carolina physician, who says it came under his personal observation in the "piney woods" of that State: "A certain girl was known to have been pretty 'thick' with a fellow named Sam. After a reasonable time, experiencing some pains which she had reasons to believe, and did verily believe, were the precursors of maternity, she sent for an old midwife who, upon examination, found *something* presenting, and proceeded with due formality to deliver, at the same time holding her peace. As soon as the young patient felt the relief of delivery, in a husky, tremulous voice she inquired, '*Does it favor Sam?*' She had only been relieved of an enormous fœcal accumulation. Thus her secret got away from her."

THE SALE AND PRESCRIPTION OF ALCOHOLIC LIQUORS BY DRUGGISTS AND PHYSICIANS IN PENNSYLVANIA.—The legislature of Pennsylvania passed, on May 11th, a high-license bill; the following is the section relating to druggists and physicians: "That druggists and apothecaries shall not be required to obtain license under the provisions of this act, but they shall not sell intoxicating liquors *except upon the written prescription of a regularly registered physician.* Alcohol, however, or any preparation containing the same, may be sold for scientific, mechanical or medicinal purposes. But no spirituous, vinous, malt or brewed liquors shall be sold or furnished to any person more than once on any one prescription of a physician; and any physician who shall willfully prescribe any intoxicating liquor as a beverage to a person of known intemperate habits shall be guilty of a misdemeanor, and upon conviction shall be fined not less than fifty nor more than five hundred dollars, and undergo an imprisonment of not less than twenty nor more than ninety days."—*Medical and Surgical Reporter, May 21, 1887.*

THE DOCTOR'S SUMMER PRACTICE.—The approach of the summer months brings to physicians reminders of the great change which has occurred in the course of medical practice during the past fifteen years. In this time there has been a constant development of, and increase in, the custom of moving to the country or seaside for the summer, so that now in cities all the wealthy families and a majority of those of moderate means leave their homes for three or four months in the year. The result is that the general practitioner in the city often finds himself almost without paying patients when the hot weather comes on, and it has come to this, that many physicians have to earn their living for the year during the fall, winter and spring months. The exodus of citizens from the cities in summer is bound to continue, for it is a practice based on good sense and good hygiene; therefore the wise physician will lay his plans accordingly. If the patient goes out of town, the doctor should go also. He should select some locality agreeable to himself and family, make it his summer home, and attend with diligence and skill to the physical ills of the exiles about him. By judicious continuance in such a course he will find that he can spend the summer pleasantly and with little expenditure of the hard earnings of the winter.—*N. Y. Medical Record.*

FATAL POISONING FOLLOWING TWO VAGINAL DOUCHES WITH CORROSIVE SUBLIMATE SOLUTION.—Fleischmann, of Prague, has reported the following case (*Therapeutic Gazette*): A perfectly healthy primipara, aged seventeen, exhibited no symptoms of kidney disease or of any other complication of pregnancy. To disinfect the vagina before labor two douches of 1 to 2,000 solution of sublimate were given, one before and one after examination by a midwife. It was noticed that a small amount of bloody mucus was expelled from the vagina after the douches. In a few hours abdominal pain, diarrhœa and a rise of temperature occurred, all the symptoms and lesions of mercurial poisoning developed, nephritis, salivation and continued diarrhœa, and, after giving birth to a living child, the patient died in coma on the ninth day after the douches were given. The pathological anatomical diagnosis made at the autopsy was "corrosive sublimate intoxication, acute nephritis, dysentery, stomatitis and pharyngitis in the stage of ulceration, parenchymatous degeneration of heart and liver, lobar and lobular pneumonia, bilateral acute cystitis." On investigation it was found that after receiving the douche the patient had left her bed and gone about her work in the ward. It is not probable that any considerable amount of fluid remained in the vagina after the douche, as all ordinary precautions were taken to secure its expulsion. It is, however, quite possible that some of the fluid may have gone into the uterus and become absorbed by any superficial lesions which had occurred in the beginning dilatation of the os uteri. It was also true that the patient had diarrhœa before the douches were given, and in his remarks upon the case Fleischmann calls attention to the danger attending the use of sublimate in puerperal patients in whom any lesion or disordered condition of the kidneys or bowels exists. He quotes the words of a well-known obstetrician, who asserts that the use of sublimate should be limited to the disinfection of the external genitalia and hands and instruments.—*N. Y. Medical Record.*

PROFESSIONAL INTERESTS.—A case was recently decided in France, the result of which shows how much keener an appreciation law takes of professional interests in France than in our own country. A journal, the *Echo de l'Est*, had thought fit to announce to its readers that Dr. Bernard, of Thiancourt, had just performed a surgical operation, in consequence of which the patient had succumbed. Dr. Bernard immediately brought an action against the journal for libel, inasmuch as it was inferred that the death of the patient was attributable to the operation. The Court of First Instance of Bar-le-Duc, looking at the matter as one of public interest, authorized the incriminated journal to substantiate the allegation. On appeal, however, the court at Nancy quashed the decision of the other court, and declined to allow the journal to plead, declaring the allegations to be groundless, adding, moreover, that "inasmuch as they were based on a fact of an essentially private nature, altogether outside public discussion, and quite independent of journalistic criticism," the journal was not warranted in making any such statement, and was condemned to pay 10,000 francs damages to the practitioner whose reputation had been assailed. It is quite refreshing in these degenerate days to see that, in one country at any rate, the reputation of a medical man is recognized as having an appreciable value. The interesting feature of the case, however, lies in the decision of the tribunal that such cases are beyond the competence of a newspaper to criticise, and are, in any event, of an essentially private nature. It would be a great boon if such a sentiment could be imported into this country. A few decisions like this would doubtless have a salutary effect in checking the by no means disinterested curiosity of scandal-mongers, who appear to consider themselves competent and at liberty to comment upon both the malady and the treatment, to the annoyance of the patient and the discomfort and even damage of the medical attendant.—*British Medical Journal*, *May 14, 1887.*

A SUCCESSFUL CASE OF CÆSAREAN SECTION.— Dr. Wm. T. Lusk reported a case of successful Cæsarean section at a meeting of the New York County Medical Society, April 18th. The operation was rendered necessary on account of deformity of the pelvis resulting from hip-disease. The abdominal incision was made through the linea alba, and extended from just below the umbilicus to a point two or three finger's breadth above the symphysis pubis. The peritoneum having been slit up, the uterus was everted by the hands over the abdomen. When the organ had been thus turned out, the intestines were placed behind it, and both the intestines and uterus wrapped in warm towels, a solution of bichloride and mercury, 1 to 10,000, being used. A rubber-tube was then placed round the uterus in order to prevent hæmorrhage. In opening the uterus an incision two inches long was made near the lower segments, and with the scissors afterwards increased to five inches. Owing to the pressure upon the vessels secured by the elastic ligature, the incision was nearly bloodless.

The child was found with the head presenting in the left occipito-anterior position, and on being extracted was in a cyanotic condition from the pressure caused by the rubber; but through the efforts of Dr. A. B. Ball it was successfully resuscitated. With the finger the membranes and placenta were readily detached from the uterine walls, and the delicate structures described by Leopold were at this time beautifully exhibited. All through the operation the intestines were held back by warm towels. The uterus remained of a cold, waxy color on account of the elastic ligature.

In closing the uterine wound, thirty-four carbolized silk sutures were employed, of which sixteen were deep and the rest superficial. In the deep sutures, he said, special pains should always be taken to avoid the mucous membrane of the cavity. The Lembert suture was used in making the superficial sutures. When the rubber-band was removed from the uterus the blood slowly returned to the pallid organ. At first it assumed a delicate rosy hue, finally a deep purple. A slight oozing was then observed at one point. The uterus was then returned to the abdominal cavity, and a drainage-tube inserted behind the organ.

Silver-wire sutures were employed to close the abdominal wound. At the end of the operation, which lasted one hour and fifteen minutes (twenty minutes of this time being taken up in endeavoring to stop the oozing referred to), the patient was in excellent condition.

For three days after the operation the temperature did not go as high as 100°. Then there was a little tympanites, and it went up above 101°; but a Seidlitz powder had the effect of promptly reducing it again. On the fifth day the drainage-tube was removed. Immediately after the operation the discharge from it was stained with blood, but it soon became colorless. Dr. Lusk said that the tube was not, in fact, needed in this case, but at the same time there was a certain feeling of security in knowing that it was in position. On the day following the removal of the drainage-tube, there was some oozing from the opening left by it. At the end of a week the abdominal sutures were removed. At this time the temperature would usually go up to about 100.5° in the evening and then fall again by morning.

On the ninth day some fluctuation was detected in the line of the abdominal wound, and a little pus was evacuated; after which the temperature became nearly normal. At the end of two weeks, however, the temperature went up suddenly to nearly 103°. Still, no trouble whatever could be discovered about the abdomen, and as the patient complained of pain in the right hip, an examination was made, which revealed an accumulation of pus in the site of the old sinuses which had given trouble during pregnancy. Since that time the patient had continued in most excellent condition. Ever since the second day, by which time she had recovered from the effects of the ether used for the operation, she had been able to take abundant nourishment. She passed her water freely, was comfortable in every way, and on the whole seemed to think it was quite an easy way of having a baby. The baby weighed ten pounds and is now doing well.—*Boston Medical and Surgical Journal.*

THE RATTLESNAKE TREATMENT FOR THE RELIEF OF PAIN.—Dr. William H. Corbusier, in an article on "The Apache-Yumans and Apache-Mojaves" (*American Antiquarian*), says: "The Apache-Mojaves sometimes resort to the Tonto medicine-men to receive the rattlesnake treatment for the relief of pain. At the agency, one day, the rattling of a snake attracted my attention, and on approaching a group of Indians, from which the sound seemed to proceed, I found a medicine-man squatting down holding a large rattlesnake in his right hand, the thumb and index finger of which encircled it close to its head, while he gently stroked its back with his left. Presently an old man advanced to him, and, saying he had a pain in his head, squatted on the ground. The medicine-man arose, and placing himself behind his patient, coiled the snake around his head, and while holding it there uttered a guttural chant, occasionally causing the snake to vibrate its rattles. He then quickly uncoiled the snake and swung it head foremost away from the man's head, at the same time making the sound *wisht*. The man then pointed to his right arm, and the medicine-man laid the snake along the limb, its head resting on the hand. He chanted again, caused it to rattle, and swung it away as before. The old man arose, and with a satisfied air walked away. Other patients succeeded him to have the snake laid on various parts of the body. After a time the medicine-man rested the snake on the ground again, and still retaining his hold of it with his right hand, put a pinch of yellow pollen into its mouth with his left and rubbed some along its belly. He then held his hand out to a man, who took a pinch of the powder and rubbed it on the crown of a boy's head. Yellow pollen treated in this manner is a common remedy for headache, and may frequently be seen on the crown of the head of men and boys."—*New York Medical Record, Feb. 26.*

GONORRHŒA AND STRICTURE.—Dr. W. S. Richardson, of Marlborough, Massachusetts, writes: "Does stricture cause irritation, or irritation cause stricture? The talk is all about stricture causing irritation. It increases irritation, no doubt. The idea seems to be that if the stricture is removed the irritation will go away. It is a common thing for a sensitive urethra to contract so that a catheter cannot be passed till the urethra becomes habituated to its presence. The constant irritation of a gonorrhœal inflammation the urethra *never* gets accustomed to till the inflammation begins to subside. In every case of gonorrhœa the inflammation is a constant stimulus to contraction. When the irritation is removed, if it is removed soon enough, there is no more contraction. If for any reason the inflammation stays for any length of time, from two to six months, there is that constant tendency to contraction—the urethra trying to eject the cause of irritation. It is likely that this continued effort will be followed by the proliferation of the elements of the submucous tissue at certain parts of the canal. This idea suggests a treatment for gonorrhœa or gleet with stricture. In the treatment of a chancroid we seek to produce a healthy unhealed surface by using one or two strong applications, and then heal this surface by continued mild applications. With hard resisting strictures there is no doubt an organic change, but if a medium-sized instrument can be passed and applications made and the irritation removed, the stubbornness of the organic (?) stricture will not be troublesome. Has success in a few cases made one observer an enthusiast over a *false* notion; or is there some sense in the above theory? There has been some trouble somewhere or there would not be so much talk about the difficulty of curing gonorrhœa and stricture."—*New York Medical Record.*

